# Effects of Ginger (*Zingiber officinale*) on the Hallmarks of Aging

**DOI:** 10.3390/biom14080940

**Published:** 2024-08-02

**Authors:** Maima Matin, Tanuj Joshi, Dongdong Wang, Nikolay T. Tzvetkov, Farhan Bin Matin, Agnieszka Wierzbicka, Artur Jóźwik, Jarosław Olav Horbańczuk, Atanas G. Atanasov

**Affiliations:** 1 Institute of Genetics and Animal Biotechnology of the Polish Academy of Sciences, Jastrzebiec, 05-552 Magdalenka, Poland; m.matin@igbzpan.pl (M.M.); a.wierzbicka@igbzpan.pl (A.W.); aa.jozwik@igbzpan.pl (A.J.); j.horbanczuk@igbzpan.pl (J.O.H.); 2 Department of Pharmaceutical Sciences, Bhimtal, Kumaun University, Nainital 263002, India; joshitanuj18@gmail.com; 3 Centre for Metabolism, Obesity and Diabetes Research, Department of Medicine, McMaster University, 1280 Main Street West, Hamilton, ON L8S 4K1, Canada; wangd123@mcmaster.ca; 4 Department of Biochemical Pharmacology and Drug Design, Institute of Molecular Biology “Roumen Tsanev”, Bulgarian Academy of Sciences, 1113 Sofia, Bulgaria; ntzvetkov@gmx.de; 5 Department of Pharmacy, East West University, Aftabnagar, Dhaka 1212, Bangladesh; farhan.bin.matin@gmail.com; 6 Laboratory of Natural Products and Medicinal Chemistry (LNPMC), Center for Global Health Research, Saveetha Medical College and Hospital, Saveetha Institute of Medical and Technical Sciences (SIMATS), Thandalam, Chennai 602105, India; 7 Ludwig Boltzmann Institute Digital Health and Patient Safety, Medical University of Vienna, Spitalgasse 23, 1090 Vienna, Austria

**Keywords:** ginger, hallmarks of aging, longevity, life-extension, lifespan, health span, metabolism, inflammation

## Abstract

Ginger (*Zingiber officinale* Roscoe) is broadly used as a traditional remedy and food ingredient, and numerous preclinical and clinical studies have demonstrated health benefits in a range of age-related disorders. Moreover, longevity-promoting effects have been demonstrated in several (preclinical) research models. With this work, we aimed to comprehensively review the reported effects of ginger and its bioactive constituents on the twelve established hallmarks of aging, with the ultimate goal of gaining a deeper understanding of the potential for future interventions in the area of longevity-extension and counteracting of aging-related diseases. The reviewed literature supports the favorable effects of ginger and some of its constituents on all twelve hallmarks of aging, with a particularly high number of animal research studies indicating counteraction of nutrient-sensing dysregulations, mitochondrial dysfunction, chronic inflammation, and dysbiosis. On this background, validation in human clinical trials is still insufficient or is entirely missing, with the exception of some studies indicating positive effects on deregulated nutrient-sensing, chronic inflammation, and dysbiosis. Thus, the existing body of literature clearly supports the potential of ginger to be further studied in clinical trials as a supplement for the promotion of both lifespan and health span.

## 1. Introduction

**Figure 1:**
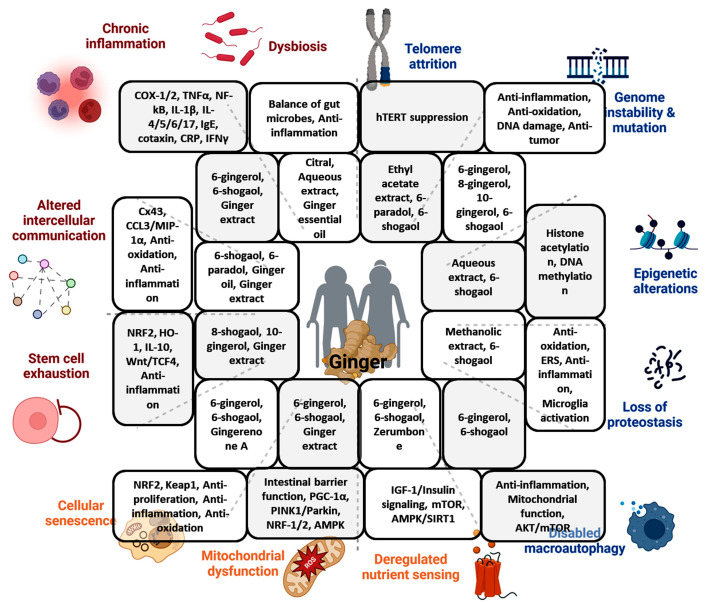
Summary of the effects of ginger and its constituents on the hallmarks of aging and the respective involved mechanisms (created with BioRender.com (accessed on 18 July 2024)).

Aging is a complex multi-component process that is defined by the gradual decline of physiological functions and homeostasis maintenance, ultimately resulting in the death of the organism. The compromised physiological functions and homeostasis result in increased susceptibility to diverse age-related neoplastic and degenerative diseases. Twelve prominent hallmarks of the aging process have been outlined, which include genomic instability, telomere attrition, epigenetic alterations, loss of proteostasis, disabled macroautophagy, deregulated nutrient-sensing, mitochondrial dysfunction, cellular senescence, stem cell exhaustion, altered intercellular communication, chronic inflammation, and dysbiosis [1,2] (Figure 1). Counteracting aging by targeting mechanisms associated with its hallmarks is of great interest, and modulation of diet and specific dietary ingredients have emerged as a powerful approach to improving health and expanding the lifespan [3,4,5].

**Figure 2:**
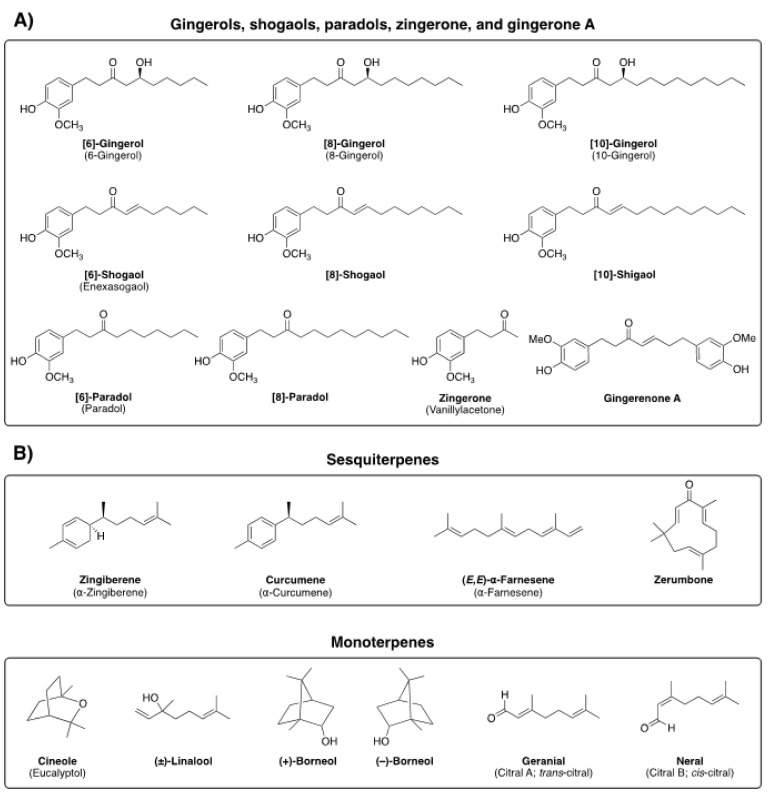
Chemical structures and common names of the major bioactive constituents of ginger, including nonvolatile (panel (**A**)) and volatile compounds (sesqui- and monoterpenes, panel (**B**)).

Ginger (*Zingiber officinale* Roscoe) is broadly used in culinary as well as utilized as a versatile remedy in the traditional folk medicine of many countries [6,7]. The major bioactive constituents of ginger are nonvolatile and include gingerols, shogaols, paradols, and zingerone, while ginger volatile oil constituents that have been identified include sesquiterpenes (zingiberene, curcumene, and farnesene) and monoterpenes (cineole, linalool, borneol, geranial, and neral) (Figure 2) [8,9]. 6-gingerol specifically is considered to be a major pungent phytochemical found in fresh ginger, whereas, in dried rhizomes, 6-gingerol is dehydrated to 6-shogaol.

There has been a broad clinical interest in verifying in patients ginger bioactivities indicated by preclinical research and traditional folk medicine, and a recent systematic study of conducted randomized controlled trials indicated robust evidence for efficacy in counteracting metabolic syndrome, for improvement of nausea and vomiting in pregnancy, for anti-inflammatory activity, for digestive function support, and for positive impact on colorectal cancer’s markers [10]. According to the European Medicines Agency’s (EMA’s) Committee on Herbal Medicinal Products (HMPC), the most established pharmacological properties of ginger rhizomes are its use in adults to prevent nausea and vomiting in motion sickness, to counteract mild symptoms of affecting the stomach or gut (e.g., bloating and flatulence), and its application in children aged six and above to combat the symptoms of motion sickness (EMA website accessed on 11 of February 2024; https://www.ema.europa.eu/en/medicines/herbal/zingiberis-rhizoma). The efficacy of ginger and its ingredients for counteracting dementia and other age-related neurological disorders has also been broadly studied [11,12]. Moreover, longevity-promoting effectiveness has been demonstrated upon the application of both ginger extract [13] and 6-shogaol [14] in a *Caenorhabditis elegans* model. Similarly, the lifespan-promoting effect of ginger was also demonstrated in *Drosophila melanogaster* models [15,16,17].

Taking into consideration that ginger is a broadly consumed medicinal plant that has shown promising potential in modulating age-related disorders and promoting healthy aging, the aim of this work is to comprehensively review the impact of ginger on the hallmarks of aging, as inspired by the two influential reviews on the latter topic by López-Otín C et al. [1,2]. Evaluation of effects by single key constituents of ginger, such as 6-gingerol, 6-shogaol, and 6-paradol, on the recently recognized hallmarks of aging is also aimed. Special attention will be given to molecular mechanisms and affected cell signaling pathways underlying ginger’s anti-aging effects, with the ultimate goal to gain a deeper understanding of the therapeutic potential of ginger and its constituents and inspire future research avenues in the area of longevity-extension interventions.

## 2. Potential of Ginger to Modulate Genomic Instability

Genomic instability represents an important contributor to aging, and it is characterized by diverse genetic lesions such as point mutations, single- and double-strand breaks, deletions, or translocations of DNA fragments, telomere shortening, and changes in chromosomal and nuclear architecture. These lesions are the result of exogenous agents (chemical, physical, or biological), as well as endogenous processes such as oxidative metabolic processes or DNA replication errors, among others. Genomic instability is counteracted by diverse mechanisms of repair and maintenance of both nuclear and mitochondrial DNA [18], and aging is associated with decreased efficiency of these protective repair mechanisms.

Several works examined in vitro or in vivo the effects of ginger or its constituents on genomic stability or parameters related to it. Ginger and some of its bioactive constituents have been shown to possess significant antioxidant and free radical scavenging activities [19], which can counteract DNA damage induced by oxidative stress. Direct comparison of the antioxidant potential of several of the major bioactive constituents of ginger, 6-gingerol, 8-gingerol, 10-gingerol, and 6-shogaol, revealed the activity of all of the studied compounds, with 6-gingerol being the least potent and 6-shogaol being most potent in the scavenging of 1,1-diphenyl-2-picyrlhydrazyl (DPPH) radicals (IC50 of 8.05 µM), as well as superoxide (IC50 of 0.85 µM) and hydroxyl radicals (IC50 of 0.72 µM) [20]. In the same work, applied at 6 micromolar concentration, all four compounds also inhibited the oxidative burst induced by N-formyl-methionyl-leucyl-phenylalanine (f-MLP) in human polymorphonuclear neutrophils. In rats, ethanolic ginger extract applied orally (at 100, 200, 300 mg/kg body weight, applied daily for 21 days) exhibited marked protective effects against bromobenzene-induced oxidative stress by boosting antioxidant defense mechanisms (increasing hepatic glutathione levels, as well as the activities of glutathione reductase, glutathione S-transferase, glutathione peroxidase, and superoxide dismutase) [21]. Similarly, co-administration of dietary ginger (1%, *w*/*w*, in parallel with oral lindane administration of 30 mg/kg bw for 4 weeks) counteracted lindane-induced lipid peroxidation, along with changes of oxygen free radical scavenging enzymes such as superoxide dismutase and catalase, reduced glutathione, and the enzymes glutathione peroxidase, glutathione reductase, and glutathione-S-transferase [22]. Moreover, clinical studies in humans also supported the antioxidant effects of ginger. Thus, it was shown that newly diagnosed patients with solid tumors receiving moderate-to-high emetogenic potential chemotherapy, when compared to placebo, had a significant increase in antioxidant enzyme blood levels (superoxide dismutase, catalase, glutathione peroxidase) and the levels of the nonenzymatic antioxidant glutathione, and also exhibited reduced oxidative marker levels (malondialdehyde and NO_2−_/NO_3−_) upon oral administration of standardized 6-gingerol (standardized 6-gingerol 20 mg/day) capsules of 5 mg twice daily, starting 3 days prior to the first cycle of chemotherapy until the fourth [23].

Anti-inflammatory properties have also been well-established for ginger, and some of its constituents, and this may also contribute to potential protective effects against genomic instability since chronic inflammation can induce DNA damage that can ultimately result in carcinogenesis [24]. In this context, aside from the mitigation of oxidative stress (that is known to be associated with chronic inflammation), the ability of ginger to counteract pro-inflammatory pathways could be of significance. In particular, research studies have indicated the inhibitory effects of ginger on pro-inflammatory enzymes such as lipoxygenase and cyclooxygenase-2 (COX-2), as well as the NF-κB pathway, among other inflammatory mediators [25].

In line with the postulated above potential of ginger to counteract DNA damage through its antioxidant and anti-inflammatory activities, a study in rats demonstrated that the pretreatment with the ginger constituent zingerone (150 mg/kg (p.o.) administrated 2 h before lipopolysaccharide challenge) abolished the increase of 8-Hydroxy-2′-deoxyguanosine (8-OHdG, DNA damage marker) levels that was associated with lipopolysaccharide-induced inflammation [26]. Similarly, the levels of the DNA damage marker 8-OHdG were suppressed upon oral aqueous ginger extract (500 mg/kg body weight, orally, for two months) application in a rat model of monosodium glutamate-induced neurotoxicity [27], upon co-application of hydro-alcoholic extract of ginger (50 mg/kg body weight, intragastrically by gavage, for six weeks) in a rat ethanol-induced kidney damage model [28], and upon hydro-alcoholic extract of ginger pretreatment (50 mg/kg body weight, intragastrically by gavage, for 10 days) in a rat model of radiation-induced kidney damage [29].

A summary of the effects of ginger and its constituents on genomic instability and the other hallmarks of aging with an outline of key supporting in vitro and animal research studies is presented in Table 1 and Table 2.

## 3. Potential of Ginger to Modulate Telomere Attrition

Telomeres are nucleoprotein structures composed of repeated sequences of DNA that cap the end of chromosomes to maintain the function of genomic stability by protecting from degradation of each cell cycle. An enzyme called telomerase, responsible for the maintenance of telomere length, functions in the cell by adding TTAGGG repeats to the 3′ end of the sequence by retrotranscription. Each time the cell divides, telomeres become shorter by losing a small amount of DNA. Over time, as a result of the progressive shortening of telomeres, chromosomes become damaged, and the cells can no longer divide, resulting in cell apoptosis. However, the telomeres can be rebuilt by telomerase to restore cell division. The largest activities of telomerase are notably observed to function in stem cells, gametes, and tumor cells. During cellular replication in somatic cells, telomere length shortens gradually, giving rise to chromosomal instability and mutagenesis, resulting in tumorigenesis [47]. Short telomere length has been linked to many age-related diseases and noncommunicable diseases, which may reflect the effects of numerous behavioral, psychosocial, and environmental factors on health status [47]. Many factors linked to certain lifestyles, such as smoking, high consumption of unhealthy diet, lack of exercise, and obesity, can increase the chance of telomere shortening, leading to premature death [48]. Risk for various cancers, such as bladder, colorectal, breast, and gastric cancer, has been associated with accelerated telomere shortening [48].

Telomeres can also be highly sensitive to damage by oxidative stress due to plenty of guanines in the telomeric repeats. This oxidative stress can be the result of reactive oxygen species (ROS) or free radicals that are produced from ATP production of mitochondria and as a by-product of aerobic metabolism [49]. Due to oxidative stress, most telomere attrition occurs during the replication of DNA, resulting in single-stranded DNA damage [50]. Along this line, a study indicated that a diet containing antioxidant omega-3 fatty acids reduced the rate of telomere shortening, whereas a lack of these antioxidants increased the rate of telomere attrition [51]. Moreover, it was demonstrated that women who consumed a diet lacking antioxidants had shorter telomeres and had moderate chances of developing breast cancer, whereas consumption of a diet rich in antioxidants had longer telomeres and a lower risk of breast cancer [52]. Cytometry studies have also shown that patients with rheumatoid arthritis (chronic inflammatory disease associated with increased oxidative stress) have accelerated shortening of telomere lengths with the increase in age, resulting in premature cellular aging [53].

Diverse dietary phytochemicals have gained interest because of their potential for the prevention of cancer because of their therapeutic efficacy, bioavailability, and safety [54]. Telomerase enzymes are one of the attractive targets for cancer prevention because telomerase-specific inhibition causes cancer cells and cancer-initiating cells to undergo apoptosis and cell senescence without any effect on somatic cells. Several phytochemicals were found to inhibit telomerase in cancer cells, including curcumin, resveratrol, silibinin, pristimerin, EGCG, and sulforaphane, among others [55]. In recent years, scientists have revealed various chemical compounds found in ginger rhizomes, which exhibited anticancer properties in many experimental models [56]. In relation to telomerase in particular, a research study revealed that the crude ethyl acetate fraction of ginger extract exhibited inhibition of the expression of human telomerase reverse transcriptase (hTERT) and c-Myc in A549 lung cancer cells in a time-(up to 72 h) and concentration-dependent (up to 64 µg/mL) manner [57]. Moreover, the result showed that from the major bioactive compounds in ginger, only 6-paradol and 6-shogaol could suppress hTERT expression and telomerase activity in A549 lung cancer cells, whereas 6-gingerol could not [58].

**Table 2:** In vivo animal research studies linked to the potential of ginger to modulate hallmarks of aging.

Hallmark of Aging	In Vivo Model and Reference	Ginger Ingredient	Dose, Mode of Administration, and Time of Treatment	Outcomes
Genomic instability	Lipopolysaccharide-induced inflammation in rats [26]	Zingerone	150 mg/kg (p.o.) administrated 2 h before lipopolysaccharide challenge	Counteracted DNA damage, quantified by measuring the DNA damage marker 8-OHdG
	Monosodium glutamate-induced neurotoxicity in rats [27]	Aqueous extract of ginger	500 mg/kg body weight, orally, for two months	Counteracted DNA damage, quantified by measuring the DNA damage marker 8-OHdG
	Chronic ethanol administration in rats [28]	Hydro-alcoholic extract of ginger	50 mg/kg body weight, intragastrically by gavage, for six weeks	Counteracted DNA damage, quantified by measuring the DNA damage marker 8-OHdG
	Radiation exposed rats [29]	Hydro-alcoholic extract of ginger	50 mg/kg body weight, intragastrically by gavage, for 10 days	Counteracted DNA damage, quantified by measuring the DNA damage marker 8-OHdG
Epigenetic alterations	C57BL/6j male mice subjected to transverse aortic coarctation surgery (to create pressure overload) [30]	6-Shogaol	Daily oral administration of 0.2 mg/kg or 1 mg/kg of 6-shogaol for eight weeks	Increases in histone H3K9 acetylation due to the transverse aortic coarctation surgery were significantly suppressed by 6-shogaol
Loss of proteostasis	Alzheimer’s disease induced in rats by using AlCl_3_ [59]	Aqueous infusion of ginger	108 and 216 mg/kg b.wt/day applied orally for 12 weeks	Reduction in amyloid plaques and improvement in cognition, psychological state as well as in the locomotor activity, as determined by rewarded alternation T-Maze test, grid floor activity cage, and accelerating speed rotarod
	MPTP-induced model of Parkinson’s disease in C57/BL mice [60]	6-Shogaol	10 mg·kg^−1^·d^−1^, orally, for 3 days	Reversal of the MPTP-induced changes in motor coordination and bradykinesia and reversal of dopaminergic neuronal loss in substantia nigra pars compacta and in stratum
	MPTP-induced model of Parkinson’s disease in C57BL/6J mice [61]	6-shogaol and ginger extract	Ginger extract was applied at 30, 100, 300 mg/kg and 6-shogaol at 10 mg/kg, by gavage, for 15 days (including the period of the MPTP exposure)	Ginger and 6-shogaol protected intestinal tight junction proteins disrupted by MPTP in the mouse colon, inhibited the increase of inducible nitric oxide synthase, cyclooxygenase-2, TNF-α, and IL-1β produced by macrophages, and suppressed the MPTP-induced enteric dopaminergic neuronal damage
Disabled macroautophagy	High-fat-diet + streptozotocin-induced diabetes in rats [62]	Gingerol-enriched ginger (18.7% 6-gingerol, 1.81% 8-gingerol, 2.86% 10-gingerol, 3.09% 6-shogoal, 0.39% 8-shogaol, and 0.41% 10-shogaol)	The gingerol-enriched ginger was applied as 0.75% wt/wt in the diet for 8 weeks	The ginger extract improved gastrointestinal health in part by improving mitochondrial function, including increased mitophagy (stimulating the removal of damaged mitochondria)
	Rats subjected to cerebral ischemia/reperfusion injury by middle cerebral artery occlusion (for 1 h, ischemia induction) followed by reperfusion for 24 h [63]	6-Gingerol	6-Gingerol was intraperitoneally injected (3 mg/kg or 6 mg/kg) 30 min before middle cerebral artery occlusion	6-Gingerol treatment induced autophagy, which counteracted the pathophysiological manifestations of the ischemia/reperfusion injury
Deregulated nutrient-sensing	Caenorhabditis elegans [13]	Ginger extract	Applied life-long at 60 μg/mL	Prolonged lifespan by 23.16% through the insulin/IGF-1 signaling pathway
	Mice with impaired insulin signaling as a result of sodium arsenite exposure [64]	6-Gingerol	50 mg/kg or 75 mg/kg body weight, daily supplied orally by gavage for 3 weeks	6-Gingerol treatment reduced elevated blood glucose levels, increased diminished plasma insulin levels, and counteracted the sodium arsenite-induced downregulation of GLUT4, IRS-1, IRS-2, PI3K, AKT, PPARγ at protein and mRNA levels
	Liquid fructose-induced adipose tissue insulin resistance in rats [65]	Alcoholic extract of ginger	50 mg/kg/day, by oral gavage, for five weeks	Attenuated hyperinsulinemia and insulin resistance (HOMA-IR) induced by liquid fructose, also reversed the increases in the Adipo-insulin resistance index and upregulated insulin receptor substrate (IRS)-1
	High-fat diet-induced obesity mouse model [66]	Steamed ginger extract (20,437.13 ± 228.75 mg/kg of 6-gingerol and 7356.67 ± 80.83 mg/kg of 6-shogaol)	40 mg/kg or 80 mg/kg daily, orally by gavage, for 8 weeks	Reduced body weight gain and fat mass accumulation, reduced liver steatosis, reversed the effect of the high-fat diet on the level of hepatic p-AMPK and SIRT1
	Obesity model induced by high-fat diet in C57BL/6N mice [34]	Zerumbone	Applied as 0.01% or 0.025% of the diet for 8 weeks	Decreased adiposity induced from the high-fat diet, increased the phosphorylation of AMPK in the white adipose tissues, and reversed the dysregulation of miR-146b as well as attenuated decrease in SIRT1 expression
	High-fat diet-fed rats [67]	6-Gingerol	200 mg/kg body weight, via oral gavage, for 2 weeks	Induced hypoglycemic effect, enhanced phosphorylated AMPK-a1, and suppressed P65 via upregulation of Sirt-6 and downregulation of resistin
Mitochondrial dysfunction	High-fat-diet + streptozotocin-induced diabetes in rats [62]	Gingerol-enriched ginger (18.7% 6-gingerol, 1.81% 8-gingerol, 2.86% 10-gingerol, 3.09% 6-shogoal, 0.39% 8-shogaol, and 0.41% 10-shogaol)	The gingerol-enriched ginger was applied as 0.75% wt/wt in the diet for 8 weeks	The ginger extract improved gastrointestinal health in part by improving mitochondrial function, including increased mitophagy and influencing the balance between mitochondrial fusion and fission
	High-fat diet-fed rats [68]	Ginger water extract	Supplemented at 0.25% or 0.5% of the diet for 14 weeks	Reduced weight gain and increased mitochondrial size and mtDNA content in rat skeletal muscle, upregulating PGC1ɑ, NRF1, and Tfam mRNA expression
	High-fat diet-induced obesity mouse model [66]	Steamed ginger extract (20,437.13 ± 228.75 mg/kg of 6-gingerol and 7356.67 ± 80.83 mg/kg of 6-shogaol)	40 mg/kg or 80 mg/kg daily, orally by gavage, for 8 weeks	Reduced body weight gain and fat mass accumulation, reduced liver steatosis, inhibited whitening of brown adipose tissue, prevented mitochondrial dysfunction, reversed the effect of the high-fat diet on the level of hepatic p-AMPK and SIRT1
	Balb/c mice [38]	Ginger water extract	2 g/kg administrated for 7 days in drinking water	Increased oxygen consumption, intrascapular temperature, and mitochondrial DNA copy number in muscle and liver, as well as increased the expression of proteins related to oxidative phosphorylation and proteins associated with the AMPK/PGC1ɑ signaling pathway in muscle, liver, and brown adipose tissue
	Aging (22-month-old) rats [69]	6-Gingerol	At 0.05 or 0.2 mg/kg, once daily via oral gavage, for 7 weeks	Counteracted age-related hepatic steatosis, increased fat oxidation, decreased fat synthesis, and improved mitochondrial function in the liver, upregulating mitochondrial marker enzymes NOX, SDH, and SIRT3
Stem cell exhaustion	*plcg1*^−/−^ mutant zebrafish embryos with hematopoiesis deficiency [70]	Ginger extract or 10-gingerol	Applied at 5 µg/mL and 2 µg/mL, respectively, from the from the late gastrulation (9–10 hpf) stage onward	Rescues the formation of hematopoietic stem/progenitor cells in hemogenic endothelium and caudal hematopoietic tissue
Chronic inflammation	Mouse model of ovalbumin-induced allergic asthma [71]	Ginger ethanol extract and aqueous extract	The ginger ethanol extract was applied at 500 mg/kg and the aqueous extract at 720 mg/kg, both for 7 days	Both extracts significantly inhibited Th2-mediated immune response, with reduction in goblet cell hyperplasia, infiltration of inflammatory cells in airways, edema with vascular congestion, and total and differential count of eosinophils and neutrophils in bronchoalveolar lavage fluid
	Male A/J mice challenged with methacholine (a model to study airway resistance in the context of asthma) [43]	8-Gingerol	The compound (at 100 μM) was aerosolized and sprayed to the mice 15 min before challenge by methacholine	Attenuated changes in central airway resistance
	High-fat diet-fed rats [72]	Ginger standardized ethanoic extract	Applied daily at 400 mg/kg by oral gavage for 6 weeks	Suppressed hepatic NFκB activation and reduced the hepatic levels of several key pro-inflammatory cytokines, including IL-6 and TNFα
	Collagen-induced arthritis in mice [73]	Ginger water extract	Orally administered at 100 and 200 mg/kg body weight, once daily from day 22 to day 35 after the induction of arthritis	Exerted an anti-arthritic effect by inhibiting the production in the serum of the IL-17, IL-4, and IFN –γ, and the paw tissue expression of MMP-1, MMP-13, and MMP-3
	DSS-induced colitis mouse model [74]	6-Gingerol	Applied once a day by gavage at 250 mg/kg for 14 days	Ameliorated bowel damage and reduced incidence of weight loss, as well as suppressed the increased serum and bowel levels of cytokines such as IL-6 and IL-17 (indicating inhibition of inflammatory responses both systematically as well as locally), and affected the cell balance of Th17/Treg
Dysbiosis	High-fat diet-fed mice [75]	Dried ginger powder	Applied at 500 mg/kg body weight, once daily via gavage for 16 weeks	Induced a decrease in low-grade inflammation, liver steatosis, and body weight, improved insulin resistance, and produced modulation in the composition of gut microbiota with an increase in the species that belong to the genus Bifidobacterium and bacteria that produce SCFA. Importantly, through fecal microbiota transplantation, it was demonstrated that the anti-obesity and health-promoting effects of ginger were transferrable
	DSS-induced colitis in mice [76]	Ginger powder	Orally administered at 500 mg/kg daily for 7 days	Mice with colitis had lower species diversity and richness, a higher abundance of pathogenic bacteria, Proteobacteria and firmicutes, an increase in Lachnospiraceae_NK4A136_group, and an increase in the abundance of Lactobacillus_murinus, Lachnospiraceae_bacterium_615, and Ruminiclostridium_sp._KB18. These increased pathogenic bacteria were decreased by ginger intake. Similarly, the DSS-treated mice showed a lower abundance of Muribaculaceae, and ginger application reversed this trend
	Apo E mice made atherosclerotic through the administration of Gubra Amylin NASH (nonalcoholic steatohepatitis) diet with L-carnitine [77]	Ginger essential oil or citral	Ginger essential oil was applied at 50 mg/kg bw/day or 100 mg/kg bw/day via oral gavage for 16 weeks, and citral was applied at 20 mg/kg bw/day via oral gavage for 16 weeks	Citral and ginger essential oil produced improvement in resistance to insulin and plasma lipid profile; decreased levels of sugar in blood and trimethylamine-N-oxide levels; inhibited plasma levels of inflammatory cytokines like interleukin-1β, and most importantly, inhibited the formation of aortic atherosclerotic lesions in the animals used in the study, while also leading to an increase in the abundance of microbes that are beneficial and decreased the abundance of microbes that are involved in the pathogenesis of cardiovascular diseases

## 4. Potential of Ginger to Modulate Epigenetic Alterations

Epigenetics is concerned with functional and heritable changes in the regulation of gene activity and expression caused by non-genetic mechanisms without altering the primary DNA sequence, which is susceptible to environmental exposures [78]. A variety of epigenetic alterations can be cell- or tissue-specific and can persist throughout life and be passed on to multiple generations [79]. Epigenetic modification is attained by specific mechanisms, involving DNA methylation, miRNAs expression, and histone modifications, and the action of non-coding RNAs, which play an important role in maintaining the stability of the genome [80]. DNA methylation is an epigenetic process involved in many cellular functions that silences expression by the addition of a small chemical modification, in particular methyl group addition, to cytosine in DNA sequence. Histone modifications regulate the physical properties of chromatin and its corresponding transcriptional state, and non-coding RNA attaches the complementary sequence, resulting in silencing of the respective gene. To regulate the gene expression, these epigenetic modifications work in coordination with each other [81].

The potential of ginger or ginger-derived compounds to impact chromatin in a cell’s nucleus and regulate epigenetic mechanisms is under investigation, whereby there are indications for effects on histone acetylation. Along this line, one research study showed that both phenylephrine and transforming growth factor-beta (TGF-β) induced histone H3K9 acetylation pathways were blocked in primary cultured cardiomyocytes and cardiac fibroblasts in rats when treated with 6-shogaol (0.3 or 1 μM pretreatment for 2 h), followed by stimulation with 30 μM phenylephrine for 48 h or TGF-β 10 ng/mL for 6 h, respectively [30]. Furthermore, upon oral administration in mice, 6-shogaol (daily oral administration of 0.2 mg/kg or 1 mg/kg for eight weeks) inhibited transverse aortic constriction-induced left ventricular hypertrophy and progression to left ventricular systolic dysfunction. All these results demonstrated the potential of 6-shogaol as a therapy for heart failure. Another in vitro histone acetyltransferase (HAT) assay was also carried out next to investigate whether 6-shogaol directly diminishes p300-HAT activity, and the results revealed that p300 HAT activity was suppressed by 6-shogaol in a dose-dependent manner (upon 30 min incubation with 6-shogaol at 0.01–100 μM) [30]. In another work, an aqueous extract of ginger (applied at a dose of 10, 30, 50, 80, and 100 μg for 24, 48, and 72 h) was demonstrated to regulate breast cancer stem cells associated-miRNAs in human breast cancer cell line MDA-MB-231, restoring of the expression of tumor suppressive miRNAs, miR-200c, miR-30a, and miR-128, as well as significantly decreasing miR-200C promoter methylation, whereby the methylation of LINE1 sequences, which correlates with global genomic DNA methylation, was increased upon ginger extract treatment, predicting an increase in genomic stability [31].

## 5. Potential of Ginger to Modulate the Loss of Proteostasis

Aging itself, as well as some age-related pathologies, such as Alzheimer’s disease, amyotrophic lateral sclerosis, Parkinson’s disease, and cataracts, are linked to impaired protein homeostasis (proteostasis), resulting in the accumulation of oxidized, glycated, misfolded, or ubiquitinylated proteins that readily aggregate as extracellular plaques or intracellular inclusion bodies [1]. Along this line, aging represents a major risk factor for the development of a broader range of impactful diseases, such as cancer, inflammation-associated disorders, diabetes, Alzheimer’s, Parkinson’s, or Huntington’s disease, which are associated with diverse dysfunctions in protein homeostasis (proteostasis) leading to the accumulation of intracellular damage [82]. Proteostasis is a process that regulates the protein metabolism within the cell for maintaining the cellular proteome and the organism itself, through a vast network of biochemical pathways. This requires regulated control of protein folding, post-translational modification, and protein degradation [83]. In healthy cells, a complex proteostasis network consisting of molecular chaperones and proteolytic machinery helps to maintain the proteostasis [82]. Highly complex interactions and intersections of proteostasis pathways might result in stress on the level of the cells and organelles, leading to the disruption of the entire network [83]. Alzheimer’s disease is an example of a complex multifactorial disease associated with protein degradation and aggregation, the pathogenesis of which involves the intersection of many biochemical pathways, and the exact mechanisms triggering the disease are still being debated [84]. In the context of this disorder, one study investigated the possible prophylactic and curative effects of an aqueous infusion of ginger (108 and 216 mg/kg b.wt/day, applied orally for 12 weeks) to assess behavioral effects on rats with induced Alzheimer’s disease [59]. The result showed that both doses exhibited a significant improvement (cognition, psychological state as well as in locomotor activity, as determined by rewarded alternation T-Maze test, grid floor activity cage, and accelerating speed rotarod, respectively) on Alzheimer’s disease features by increasing acetylcholine and decreasing acetylcholinesterase in the brain. However, it also showed the high dose of ginger (216 mg/kg) exhibited a better effect than the low dose (108 mg/kg). Histopathological findings in brain cells showed closer to the control group phenotype, and the amyloid plaques disappeared [59]. The second most common neurodegenerative disorder after Alzheimer’s disease is Parkinson’s disease. Its pathogenesis is mainly characterized by the progressive loss of dopaminergic neurons of the substantia nigra pars compacta of the midbrain and the abnormal accumulation and aggregation of the protein alpha-synuclein in the form of Lewy bodies and Lewy neurites. Thereby, the protein misfolds to give rise to beta-sheet-rich amyloid fibrils. This phenomenon leads to a deficiency of dopamine with associated symptoms such as posture rigidity, bradykinesia or slow movement, instability, and resting tremors [85]. Current treatment of this disease includes levodopa and dopamine agonists which provide systematic relief accompanied with severe side effects. In contrast, natural phytochemicals have obtained promising attention as potential candidates for the therapeutic and preventative effect of Parkinson’s disease, given their multitarget pharmaceutical mechanism of actions and good safety profile; ginger and its bioactive compounds, in particular, have shown a beneficial effect in multiple Parkinson’s disease models [86,87,88]. One study, for example, demonstrated that the pungent compound of ginger, 6-shogaol (10 mg·kg^−1^·d^−1^, orally, for 3 days), protected against MPTP- (1-methyl-4-phenyl-1,2,3,6-tetrahydropyridine) or MPP^+^- (metabolite of MPTP) induced movement impairment and neuronal damage (reversal of dopaminergic neuronal loss in substantia nigra pars compacta and in stratum) by regulating microglial activation and downstream pro-inflammatory factors in a mouse (C57/BL) model of Parkinson’s disorder [60]. Another similar work showed that ginger and 6-shogaol (ginger extract was applied at 30, 100, 300 mg/kg and 6-shogaol at 10 mg/kg, by gavage, for 15 days, including the period of the MPTP exposure) restored the disruption of intestinal barrier and enteric dopaminergic neurons in an MPTP-injected mouse Parkinson’s disease model by impeding the processes of inflammation and apoptosis, suggesting that they may have potential application to attenuate the gastrointestinal dysfunction in patients with Parkinson’s disorder [61].

In general, different lines of evidence have pointed out the correlation between aging and the accumulation of oxidatively damaged proteins, lipids, and nucleic acids. Oxidatively modified proteins increase along with age, as well as the manifestation of age-related diseases associated with oxidatively modified proteins also increases [89,90]. The accumulation of these oxidized proteins during aging randomly damages the DNA responsible for protein synthesis, which also controls the generation of reactive oxygen species, proteolytic activities to degrade oxidized proteins, and antioxidant defense mechanisms [91]. Protein oxidation serves as a useful marker for assessing oxidative stress in vivo. Proteins are oxidized when there is an imbalance of reactive oxygen species and antioxidant defenses. One research study investigated the effect of ginger on the occurrence of oxidative stress in the small intestine of streptozotocin-induced diabetic rats. The results revealed that consumption of ginger (applied as a ginger powder as 5% of the daily food for 6 weeks), l, a potent oxidant, improved diabetes-induced oxidative stress in diabetic rats with attenuation of lipid peroxidation and protein oxidation and increased catalase activity [92]. Patients suffering from diabetic conditions produce oxidative stress, and various tissues are damaged [93,94]. Ginger showed hypoglycemic effects and helped to delay the development of diabetes mellitus in several studies, highlighting its antidiabetic potential [95]. One of the relevant studies was undertaken to see the dose-response effect of ginger in the inhibition of oxidative stress and clastogenicity in streptozotocin-induced diabetic rats. The results of this study in vivo showed that feeding the rats with ginger at different concentrations, such as 0.5%, 1%, and 5% of the total diet, significantly lowered the glucose, cholesterol, and triglycerides in streptozotocin (30 mg/kg)-induced diabetic rats in a dose-dependent manner. It was noted that serum glucose levels did not reach up to normal levels when ginger was fed to diabetic rats, but a reduction was observed in a dose-dependent manner [96]. Similar results were also reported in other studies in streptozotocin-induced diabetic rats, with ginger administration leading to a significant decrease in fasting glucose levels [97] and delay of diabetic cataracts [98]. In another in vitro study, the therapeutic potential of methanolic extract from ginger was investigated against glycation and oxidative stress, along with several other methods of anti-glycating and anti-aggregation test of glucose-exposed bovine serum albumin in the presence and absence of the ginger extract [32]. Taking into consideration the known pro-inflammatory action of advanced glycation end-products [99], the results of the latter study indicated that the ability of the ginger extract to inhibit glycation and heat-induced protein denaturation could be a possible contributing factor to its anti-inflammatory activity. In more detail, in the discussed work, the ginger extract (prepared by soaking of hundred grams of ginger powder in 1 L of 97% methanol) was effective in vitro against heat-induced albumin denaturation, and the percent of inhibition increased along with the increased concentration of the ginger extract that was applied in the concentration range 100–600 µg/mL [32]. A similar result was reported in another study, which showed that ginger extracts (at 125–500 µg/mL) possessed high anti-inflammatory activity by inhibiting the heat-induced albumin denaturation [100]. The highest percentage of inhibition (66%) was exerted by 50% of ethanolic extract, followed by 70% and 90% of ethanol extracts (65% and 63%, respectively), and moderate inhibition activity was observed with 80% ethanol extract (58%) [100].

While the research described above indicates that ginger counteracts protein modification and denaturation in the context of conditions such as diabetes and inflammation, another work indicated that activation of endoplasmic reticulum (ER) stress, associated with accumulation of unfolded or misfolded proteins in the ER lumen and unfolded protein response, underlies the mode of the killing of cancer cells exposed to ginger extract [101]. ER is, in general, responsible for the biosynthesis, folding, maturation, and stabilization of proteins and represents an organelle of major importance for proteostasis maintenance. A variety of pathophysiological conditions disrupt the protein folding capacity, resulting in the accumulation of unfolded, misfolded or toxic proteins in this organelle, with the process being termed “ER stress” [102]. Previous research has demonstrated that ER dilation is a characteristic response to ER stress, which develops as a result of the buildup of misfolded proteins in the ER lumen. To overcome the imbalance of ER protein folding capacity, cells have a signal transduction pathway called unfolded protein response (UPR) that becomes triggered and integrates ER stress signals to relieve the ER stress resulting in the re-establishment of the proteostasis balance. The UPR is controlled by three ER-transmembrane receptors, namely inositol-requiring enzyme 1α (IRE1α), pancreatic endoplasmic reticulum kinase (PERK), and activating transcription factor 6 (ATF6) [103]. When these ER transmembrane receptors detect the onset of ER stress, it initiates the unfolded protein response, whereby the prolongation of the stress causes apoptotic death of the cell [102]. In the above-mentioned study with ginger extract-exposed cancer cells [101], expressions of important ER stress markers such as polyubiquitinated proteins, Bip and CHOP were probed, and it was found that the ER stress proteins were increased when treated with increasing ginger extract concentrations. Moreover, this work indicated that ginger extract induced the paraptosis mode of death in cancer cells [101].

## 6. Potential of Ginger to Modulate Disabled Macroautophagy

Macroautophagy represents a process in which cytoplasmic content is incorporated in double-membrane vesicles, autophagosomes, which fuse with lysosomes and mediate degradative processes that are of essential importance for cellular homeostasis and adaptation to stress conditions such as energy- or nutrient-starvation. Autophagy declines with age advancement, and impaired autophagy is involved in the pathogenesis of a number of age-related diseases, while interventions stimulating autophagy have been shown to promote longevity [104].

In the context of autophagy modulation, one research work examined the effect of gingerol-enriched ginger extract in diabetic rats [62]. In this study, dietary application of gingerol-enriched ginger (at 0.75% of the total diet for 8 weeks) induced improved glucose tolerance and increased pancreatic insulin content in the diabetic animals, which was in part explained by improved intestinal integrity and mitochondrial dysfunction [62]. Along this line, the gingerol-enriched ginger supplementation was associated with an increase in the intestinal tissue expression of tight junction (Claudin-3) and antioxidant capacity (SOD1) genes in the intestine, and diminished expression of genes associated with mitochondrial fusion (MFN1), fission (FIS1), biogenesis (PGC-1α, TFAM), mitophagy (the removal of dysfunctional mitochondria through autophagy; LC3B, P62, PINK1), and inflammation (NF-κB) [62]. In another work, 6-gingerol (upon pretreatment with 10–40 μM 6-gingerol for 24 h) was also shown to induce autophagy in human umbilical vein endothelial cells, resulting in their improved survival from apoptosis induced by hydrogen peroxide exposure, with the findings suggesting that the compound may be beneficial in the prevention of atherosclerosis (a pathological process promoted by endothelial apoptosis) [33]. Moreover, 6-gingerol also displayed potential for the treatment of ischemia-reperfusion injury by a mechanism involving inhibition of NLRP3 inflammasome and apoptosis through TRPV1/FAF1 complex dissociation-mediated autophagy [63].

Several compounds from ginger also displayed anticancer cell-specific effects mediated through autophagy induction. Thus, 6-shogaol was shown to induce autophagy associated with inhibiting the AKT/mTOR pathway in the human non-small cell lung cancer A549 cells and in cervical carcinoma HeLa and SiHa cells [105,106]. 6-shogaol was also shown to enhance the anticancer effects of several chemotherapeutics via increase of apoptosis and autophagy in colon cancer cells under hypoxic/aglycemic conditions [107], and to suppress breast cancer cells and stem cell-like spheroids by modulation of the Notch pathway and autophagic cell death [108]. Similarly, anticancer cell-specific effects mediated through autophagy induction were reported for other ginger constituents. Thus, 6-gingerol induced caspase-3 dependent apoptosis and autophagy in cancer HeLa cells [109], zingiberene suppressed in vivo (xenografted tumors, zingiberene applied at 10, 20, and 40 mg/kg) and in vitro (growth inhibition IC_50_ of 20 µM) human colon cancer HT-29 cell growth via autophagy induction with inhibition of PI3K/AKT/mTOR pathway and caspase-2 [110], and zerumbone activated apoptosis and autophagy in human hormone-refractory prostate cancers [111].

## 7. Potential of Ginger to Modulate Deregulated Nutrient-Sensing

Nutrients are simple organic chemical compounds in food that are involved in biochemical reactions producing energy for the maintenance of health and the ability to grow, move, and reproduce [112]. Nutrition has scientifically been proved by various studies as one of the key factors for healthy aging. Imbalance in diet can cause deficiency- or excess-related diseases such as blindness, anemia, scurvy, rickets, and goiter, as well as common chronic systemic diseases associated with aging such as cardiovascular disease, diabetes, and osteoporosis [113]. It has been demonstrated that caloric restriction can be a successful dietary approach to increase the lifespan in a healthy manner [114]. Alterations in nutrient sensing pathways have gained increased attention in the field of aging as they can be regulated both pharmacologically and with dietary interventions [115]. Nutrient sensing pathways become deregulated (a hallmark of cellular aging) and lose their effectiveness along with age. Metabolic processes are causing stress to cells. Vigorous metabolic activity and changes in nutrient composition can cause cells to age faster. Metabolism and its by-products over time damage the cells through oxidative stress, ER stress, mitochondrial dysfunction, and calcium signaling [116]. Some of the key protein mediators/pathway hubs that regulate some of the nutrient-sensing pathways in the context of aging are the insulin-like growth factor 1 (IGF-1), mammalian target of rapamycin (mTOR), AMP-activated protein kinase (AMPK), and sirtuins [115,117]. Nutrient-sensing pathways play an important role in the regulation of protein synthesis, cell cycle, DNA replication, autophagy, stress response, and glucose homeostasis [115,118].

One of the most discussed topics in gerontology is the role of IGF-1/insulin signaling in the regulation of longevity. Accumulating evidence suggests that downregulated IGF-1 significantly prolonged the lifespan in vertebrate and invertebrate models [119]. Several in vitro and in vivo studies demonstrated favorable modulation of IGF-1/insulin signaling by ginger or some of its bioactive ingredients. Thus, a longevity-promoting effect of ginger (lifespan extension of 23.16% upon application of 60 µg/mL ginger extract) via the insulin/IGF-1 signaling pathway was demonstrated in a *Caenorhabditis elegans* model [13]. Suppression of IGF-1 was also observed in humans, in particular in obese women diagnosed with breast cancer, who received orally four capsules containing 750 mg of ginger flour for 6 weeks [120]. Multiple studies also demonstrated the beneficial effects of ginger on insulin signaling in the context of diabetes. Thus, ginger extract enhanced the differentiation of mouse 3T3-L1 preadipocytes (in vitro model for anti-diabetic action), and the isolated active constituent 6-gingerol upregulated insulin-sensitive glucose uptake [121]. Moreover, 6-gingerol (50 mg/kg or 75 mg/kg body weight, daily supplied orally by gavage for 3 weeks) was also shown to display anti-hyperglycemic activity and to attenuate sodium arsenite-induced impairment of insulin signaling in mice [64]. Along the same line, orally applied ginger extract (50 mg/kg/day, for five weeks) attenuated hyperinsulinemia and insulin resistance induced by liquid fructose supplementation in rats [65], and human trials (Table 3) have consistently demonstrated improvement of blood glucose levels (key target of insulin action) in type 2 diabetes patients supplemented with ginger [122]. As one example, Arablou et al. performed a double-blind, placebo-controlled clinical trial with 70 type 2 diabetes patients who were allocated randomly to receive a placebo or 1600 mg ginger (equaling two capsules) for 12 weeks, and the ginger-receiving group displayed both improved metabolic parameters (reduction of fasting glucose, HbA_1C_, insulin, HOMA, triglycerides, and total cholesterol) as well as reduction in the level of the inflammatory markers CRP and PGE_2_ [123]. Similarly, a randomized, placebo-controlled clinical trial with 103 individuals demonstrated that daily intake of 1.2 g of ginger (two capsules, 600 mg ginger powder in each) for 90 days resulted in a greater reduction in the blood glucose and total cholesterol in comparison to the placebo group [124].

mTOR is a key coordinator of eukaryotic cell growth and metabolism with input from environmental signals, including growth factors and nutrients (notably, being activated by high amino acid concentrations) [127]. Suppression of mTOR activity is one of the most established interventions resulting in extended lifespan in a wide range of different species [128]. Several research works reported the modulation of mTOR by ginger and some of its bioactive constituents. Thus, supplementation with steamed ginger extract (40 mg/kg or 80 mg/kg daily, orally by oral gavage, for 8 weeks) was demonstrated to inhibit mTOR activation and fat accumulation induced by high-fat diet in mice [66]. Several studies also reported that 6-shogaol inhibited mTOR and induced cell death in non-small cell lung cancer and cervical carcinoma cells [105,106]. Similarly, zingerone (applied at 15 and 20 µM for 24 h) and zerumbone induced apoptosis and inhibition of the mTOR signaling in human prostate cancer PC-3 cells [129] and oral squamous cell carcinoma cells [130], respectively. 6-Gingerol was also reported to act as an mTOR inhibitor, and the compound was shown to counteract neuroinflammation and ischemic brain injuries through suppression of Akt/mTOR/STAT3 signaling in microglia cells [131].

AMPK senses low energy status in cells by detecting high levels of adenosine monophosphate (AMP), which is a product of the energy production happening through ATP hydrolysis. The activation of AMPK by high AMP levels activates catabolic processes aiming to restore energy homeostasis (to generate more ATP) and, among other effects, increases cellular NAD^+^ levels, which in turn leads to the activation of sirtuins, NAD^+^-dependent family of deacetylases, the activation of which is also viewed as a key anti-aging mechanism [132,133], along with the suppression of mTOR and IGF-1/insulin signaling [117]. Similarly to modulating mTOR and the IGF-1/insulin signaling (as discussed above), many research works have also studied the effects of ginger and some of its compounds on AMPK and sirtuins. Thus, it was demonstrated that steamed ginger extract supplementation reduces liver steatosis and adipocyte metabolic dysfunction through AMPK-SIRT-1 activation in a high-fat diet (HFD)-induced obesity mouse model [66]. Similarly, zerumbone, a ginger sesquiterpene (applied as 0.01% or 0.025% of the diet for 8 weeks), counteracted the obesity induced by high-fat diet in C57BL/6N mice through activation of AMPK and sirtuins (SIRT1) [34] and 6-gingerol (20 μM, for 8–9 days) promoted browning (favorable metabolic phenotype) in mouse 3T3-L1 adipocytes with the upregulation of SIRT1 and p-AMPK/AMPK together with several other biomarkers [35], as well as it exhibited hypoglycemic effect in high-fat diet-fed rats that was associated with increased phosphorylated (activated) AMPK-α1 and sirtuin-6 [67].

## 8. Potential of Ginger to Modulate Mitochondrial Dysfunction

Mitochondria exhibit the capability to produce ROS alongside their electron transport process. While ROS at low concentrations function as physiological signals, their excessive generation in response to stress exerts a toxic influence, leading to detrimental effects on mitochondrial components and gradual impairment of mitochondrial function. To counterbalance this potential harm, a range of endogenous antioxidant defense mechanisms come into play. These encompass antioxidant proteins like glutathione peroxidase 1, superoxide dismutase 2, and peroxiredoxin 3/5, which are regulated by transcription factors such as Nuclear factor (erythroid 2-like)-2 (Nrf2) [134]. Additionally, small molecules including coenzyme Q and glutathione contribute to mitigating the oxidative stress within mitochondria [135]. As individuals age, there is a decrease in the mitochondrial membrane potential, resulting in diminished ATP generation. This decline is further associated with the activation of molecules associated with inflammation and ROS, ultimately culminating in cellular demise [136]. A notable hallmark of aging mitochondria is the surge in somatic point mutations and large deletions in mtDNA, leading to dysfunction. These mtDNA mutations, often arising due to oxidative damage in proximity to ROS sources, play a central role in mitochondrial dysfunction. The Mitochondrial Free Radical Theory of Aging (MFRTA) posits a cycle where oxidative damage to mtDNA initiates respiratory chain protein dysfunction, elevating ROS production, thereby perpetuating mitochondrial dysfunction [137]. Mitochondrial dynamics, involving fusion, fission, and mitophagy, are crucial for cellular functions. One recent study indicated that Gingerol Enriched Ginger (GEG, 18.7% 6-gingerol, 1.81% 8-gingerol, 2.86% 10-gingerol, 3.09% 6-shogoal, 0.39% 8-shogaol, and 0.41% 10-shogaol) applied as 0.75% wt/wt in the diet for 8 weeks could enhance insulin production, improve intestinal integrity, and counteract mitochondrial dysfunction in high-fat-diet and streptozotocin-induced diabetes in rats [62]. GEG supplementation boosted intestinal barrier function by increasing Claudin-3 expression and curbing mitochondrial oxidative stress and inflammation. Along this line, GEG influenced the balance between mitochondrial fusion and fission, triggering mitophagy through the PINK1/Parkin pathway, and regulating PGC-1α and TFAM expression [62]. The findings suggest that GEG has the potential to alleviate type 2 diabetes-induced intestinal dysfunction, enhance insulin production, and boost beta cell number (as evidenced by the observed increase in insulin-producing beta cells detected through insulin-immunohistochemistry staining) [62].

Mitochondrial dysfunction in skeletal muscle is linked to various transcription regulators involved in mitochondrial biogenesis. Peroxisome proliferator-activated receptor gamma coactivator-1-a (PGC-1α) increases nuclear respiratory factor (NRF)-1 and NRF-2, leading to mitochondrial DNA transcription [138]. A study showed that ginger ethanol extract (applied at 100 mg/kg or 200 mg/kg daily by oral gavage for 10 weeks) improves insulin resistance by enhancing AMPK-α1 expression in skeletal muscle in high-fat, high-carbohydrate diet-fed rats [36]. Additionally, 6-gingerol (50–150 µM, applied up to 24 h) promotes AMPK-α1 activity, PGC-1α mRNA expression, and mitochondrial biogenesis in L6 myotubes [36]. One study also sheds light on the putative anti-tumor effects of 6-gingerol, focusing on the promotion of mitochondrial biogenesis in tumor-infiltrating CD8^+^ T cells and their cytotoxic effect [37]. The latter study indicates that 6-gingerol (applied at 25–50 mg/kg, i.p., for 16 days) inhibits tumor growth by 33–37% in a Lewis lung carcinoma xenograft mouse model. The ginger extract increased mitochondrial mass in CD8^+^ T cells in vivo as well as in vitro in the CTLL-2 cells model (upon application at 5 and 10 mg/mL for 24 h), enhancing their cytotoxicity against cancer cells [37]. Further analysis with the active ingredient 6-gingerol showed that it promotes mitochondrial function and biogenesis, potentially contributing to the CD8^+^ T cells’ anti-tumor effects. These findings offer a deeper understanding of the mechanisms behind ginger’s anti-tumor properties and its potential application in cancer management [37].

Muscle mitochondrial dysfunction is associated with obesity-related metabolic disorders [139]. Ginger water extract supplementation (at 0.25% or 0.5% of the diet for 14 weeks) in high-fat diet-fed rats reduced weight gain and increased mitochondrial size and mtDNA content in rat skeletal muscle, upregulating PGC1α, NRF1, and Tfam mRNA expression [68]. The ginger extract supplementation also raises serum HDL-C levels and contains compounds like 6-gingerol, known for antioxidant, anti-inflammatory, and anti-obesity effects [140]. Another research study shows that the addition of Steamed Ginger Extract (SGE) (applied at 40 mg/kg or 80 mg/kg daily, via gavage, for 8 weeks) inhibited the whitening of brown adipose tissue in supplemented mice groups by preventing increased lipid deposition and mitochondrial dysfunction [66]. This resulted in the restoration of reduced mitochondrial DNA, enzyme activities, and thermogenesis genes like UCP1. In liver and adipose tissues, SGE controlled ROS amplification and mitochondrial dysfunction induced by hyper-nutrient conditions through the mTOR-SREBP1-ER stress pathway. AMPK-SIRT1 activation by SGE was found to regulate cellular pathological signaling for liver and adipocyte dysmetabolism, mitigating liver steatosis and adipocyte metabolic issues while addressing ER and mitochondrial redox-linked disorders [66].

Another relevant study showed that ginger water extract, particularly at a dose of 2 g/kg administrated for 7 days, increased oxygen consumption, intrascapular temperature, and mitochondrial DNA copy number in muscle and liver in Balb/c mice [38]. This outcome was accompanied by an increase in the expression of proteins related to oxidative phosphorylation and proteins associated with the AMPK/PGC1α signaling pathway in muscle, liver, and brown adipose tissue. In HepG2 cells, the ginger extract (applied at 2.5 and 5 mg/mL for 3 days) enhanced mitochondrial mass, mtDNA copy number, ATP production, and activities of mitochondrial respiratory chain complexes. The study suggests that the extract promotes mitochondrial biogenesis and function through the AMPK-PGC1α signaling pathway, with 6-gingerol being the likely active component, while 6-shogaol showed inhibitory effects on cell viability [38]. It is also demonstrated that 6-gingerol (applied at 2 µM) acts as a PPARδ ligand, enhancing PGC-1α expression and muscle mitochondrial biogenesis [141].

Hepatic fat accumulation is controlled by factors like lipid processing and oxidative stress. 6-gingerol showed promise in reducing liver fat in models of non-alcoholic fatty liver disease. Along this line, one study showed that in aging (22-month-old) rats, 6-gingerol treatment (0.05 or 0.2 mg/kg, once daily oral gavage for 7 weeks) counteracted age-related hepatic steatosis, increased fat oxidation, decreased fat synthesis, and improved mitochondrial function in the liver, upregulating mitochondrial marker enzymes such as NOX, SDH, and SIRT3 [69]. These effects were achieved by affecting specific proteins and microRNAs related to fat metabolism. The results suggested that 6-gingerol application could be a valuable approach to combat age-related hepatic steatosis, potentially aiding in treating metabolic disorders [69].

## 9. Potential of Ginger to Modulate Cellular Senescence

Cellular senescence is characterized by a permanent cell cycle arrest state where cells are unable to divide and no longer proliferate. Senescent cells exhibit distinct features such as alterations in appearance and metabolism, modifications in chromatin structure, and shifts in gene expression patterns [142,143]. Senescence plays a crucial role in the age-related decline of tissue regenerative potential. This notion is supported, for example, by evidence from BubR1 progeroid mice, where progenitor cell populations in skeletal muscle and fat tissues show a high susceptibility to cellular senescence [144]. Apart from inducing a persistent growth arrest in stem cells, senescence can also disrupt the local stem-cell niche through the secretion of a group of pro-inflammatory and pro-growth factors known as the Senescence Associated Secretory Phenotype (SASP) [145] or Senescence Messaging Secretome [146]. Moreover, additional SASP-related mechanisms may contribute to tissue dysfunction. As an example, senescent cells release proteases that can continuously affect the structure and organization of tissues by breaking down membrane-bound receptors, proteins, or surrounding components within the tissue environment [147]. In Blagosklonny’s theory of ‘hyperfunction,’ aging is conceptualized as a quasi-program resulting from the intricate processes that take place during early life’s development and growth [148]. For instance, when growth arrest happens, certain nutrient-sensing pathways, such as mTOR, continue to operate. However, instead of promoting cell proliferation and growth as they did before, these pathways now trigger cellular senescence [148]. As human lifespans increase, age-related diseases become more prevalent in the aging population. Some of these diseases, such as sarcopenia [149], osteoarthritis, idiopathic pulmonary fibrosis, atherosclerosis, and Alzheimer’s disease exhibit features related to the activation of senescence [150].

Ginger might hold promise as a potential dietary additive to counteract muscle aging due to its antioxidant properties. Such effects of ginger can be attributed to 6-gingerol and 6-shogaol, which both were shown to exert antioxidant properties through the Nrf2 signaling pathway [151]. Nrf2 dissociates from its inhibitory protein partner, Kelch-like ECH-associated protein 1 (Keap1), leading to Nrf2 translocation into the nucleus. Once in the nucleus, Nrf2 binds to the antioxidant response element (ARE) and induces the transcription of antioxidant genes [152]. One research study investigated the effects of 6-gingerol and 6-shogaol, present in two different standardized ginger extracts (GE1 and GE2), on myoblast cells in culture. The composition of these extracts differed significantly in their content of 6-gingerol and 6-shogaol, likely attributed to variations in the extraction conditions (GE1 contained 6-gingerol and 6-shogaol at concentrations of 289.531 μg/mL and 15.466 μg/mL, while GE2 contained 181.257 μg/mL and 63.425 μg/mL, respectively). The findings revealed that treatment with 6-gingerol and 6-shogaol enriched extracts (at 100 or 300 μg/mL, for 24 h) led to morphological changes in senescent (population doubling = 21) primary human myoblasts, resembling the morphology of young myoblasts [39]. These extracts exhibited an aging-reversal effect on myoblast cells by reducing the expression of senescence-associated β-galactosidase, a marker of replicative senescence. The study further demonstrated that both 6-gingerol and 6-shogaol, despite their distinct chemical structures, produced similar effects on myoblasts when used in combination with the standardized ginger extracts. Moreover, treatment with 6-gingerol and 6-shogaol enriched extracts influenced the progression of the cell cycle in myoblast cells, with cell cycle arrest observed during the G0/G1 phase. The study suggested that the expression of the Ki67 protein, crucial for cell proliferation, was lower during the G0/G1 phase, indicating a reduction in proliferative capacity in the myoblast cells. Taken together, these findings demonstrate the potential of 6-gingerol and 6-shogaol, present in ginger extracts, to protect against cellular senescence and promote myoblast differentiation.

Replicative senescence, caused by telomere shortening, is a tumor-suppressor mechanism. However, cancer cells can evade this process by reactivating telomerase. As already mentioned in the chapter “Potential of ginger to modulate telomere attrition”, a study by Kaewtunjai et al. demonstrated that ginger extract suppressed hTERT expression, inhibited telomerase, and induced cellular senescence in A549 lung cancer cells [58]. Further investigations confirmed that prolonged treatment of A549 cancer cells with sub-cytotoxic ginger extract doses (5 and 10 μg/mL) resulted in decreased telomere length, induced cellular senescence, and reduced clonogenicity. Additionally, the active compounds within the ginger extract, 6-paradol, and 6-shogaol, showed a favorable safety profile in rats and exhibited an ancillary chemoprotective effect against the formation of liver micronuclei induced by diethylnitrosamine (DEN) [58]. The latter finding is of relevance in the context of aging since micronuclei (generally linked to chromosome instability, mutagenesis, and genome rearrangements) are frequently observed during cellular senescence [153].

Senescence is a complex phenomenon with both beneficial and harmful effects depending on the physiological context and the age of the organism. In young organisms, senescence plays a positive role in processes like wound healing, tissue remodeling, embryonic development, and tumor suppression [154]. However, in aging tissues, the accumulation of senescent cells can lead to premature aging and age-related disorders, including chronic inflammation, tumorigenesis, liver fibrosis, atherosclerosis, insulin resistance, and neurodegenerative disorders [150]. To address this issue, senotherapies, such as senolytics and senomorphics, have emerged, aiming to selectively target and eliminate senescent cells to mitigate their adverse impact. In this context, a ginger ingredient, gingerenone A (applied for 48 h at 10 or 20 μM), is identified as a senolytic compound as it effectively and selectively reduced the viability of senescent cells (WI-38 human fibroblasts rendered senescent by exposure to ionizing radiation), enhanced the expression of cleaved caspase-3 (a marker of apoptosis), and decreased levels of the anti-apoptotic protein Bcl-XL [40]. Additionally, gingerenone A treatment led to a reduction in secreted levels of pro-inflammatory factors IL-6, CCL2 (MCP-1), and interferon γ-induced protein 10 (IP-10) while increasing anti-inflammatory cytokines IL-10 and IL-13. Interestingly, treatment with 72.4 nM of 6-shogaol resulted in a notable increase in the number of proliferating cells, indicating its ability to promote cell proliferation at that application dose [40].

According to global cancer statistics from 2020, breast cancer is the most frequently diagnosed cancer in women and the leading cause of cancer-related deaths [155]. It accounts for 11.7% of all cancer cases. Various markers, including estrogen receptor, progesterone receptor, human epidermal growth factor receptor 2 (HER2), and Ki67, are used for its diagnosis [156]. One study demonstrated that gingerenone A had a higher cytotoxic impact (IC50 values ranging from 50 to 76 μM) on breast cancer cells compared to normal breast cells [157]. Gingerenone A also demonstrated a dose-dependent inhibition of cell viability in various breast cancer cell lines (SKBR3, MCF7, and MDA-MB-231). The study also revealed that gingerenone A upregulated senescence-associated gene expressions and oxidative stress were identified to play a crucial role in the antiproliferative effects of the compound on breast cancer cells [157].

## 10. Potential of Ginger to Modulate Stem Cell Exhaustion

Stem cell exhaustion represents a qualitative and quantitative decline of stem cells, that is characteristic to senescent tissues and organs and is regarded as one of the driving forces of aging in general [1]. Several works explored the effects of ginger and some of its bioactive compounds on stem cells. One study investigated exosome-like nanoparticles from four edible plants, including ginger, demonstrating that upon oral delivery (1 mg per mouse via gavage for 6 h), these nanoparticles are taken up by intestinal macrophages and stem cells [158]. Importantly, the ginger nanoparticles activated Nrf2, enhanced the expression of the antioxidant gene heme oxygenase-1 and the anti-inflammatory cytokine IL-10, as well as enhanced the activity of the Wnt/TCF4 pathway, which is known to be of critical importance for stem cell maintenance [159].

Another study investigated the effect of 8-shogaol on fibroblast-like synoviocytes (sometimes referred to in the literature as “synovium-derived mesenchymal stem cells” [160]), a cell type with a crucial role in rheumatoid arthritis development [161]. In this work, 8-shogaol application resulted in significant inhibition of TNF-α-, IL-1β-, and IL-17-mediated inflammation and migration of fibroblast-like synoviocytes derived from rheumatoid arthritis patients (applied at 5 µM and 10 µM) as well as in 3D synovial culture system. Further study of the associated mechanism of action revealed that the compound suppressed IKK, Akt, and MAPK signaling pathways [161] via direct inhibition of transforming growth factor-β (TGF-β)-activated kinase 1 (TAK1), signaling protein of key importance for both inflammatory responses and mesenchymal stem cell proliferation [162,163]. Reflecting these molecular events, 8-shogaol treatment reduced paw thickness and improved walking performance in the adjuvant-induced arthritic rat model.

It was also demonstrated that ginger extract or 10-gingerol treatment (applied at 5 µg/mL and 2 µg/mL, respectively, from the late gastrulation (9–10 hpf) stage onward) could rescue the formation of hematopoietic stem/progenitor cells in hemogenic endothelium and caudal hematopoietic tissue in *plcg1*^−/−^ mutant zebrafish embryos with hematopoiesis deficiency [70]. The latter research suggests that ginger may act towards the promotion of hematopoiesis.

## 11. Potential of Ginger to Modulate Altered Intercellular Communication

Effective intercellular communication is a fundamental requirement for maintaining the balanced functioning of cells, tissues, and organs within a healthy organism. When this communication breaks down, it has been linked to the aging process and the emergence of various age-related disorders. This disrupted communication is considered a key aspect of aging, along with other factors like cellular senescence, mitochondrial dysfunction, and genomic instability [2]. A notable example of communication disruption during aging is “inflammaging”, a persistent low-grade inflammatory state observed in older individuals. This state arises due to continuous stimulation of the immune system by various signals, including misdirected molecules and the presence of senescent cells. Intercellular communication mediated by various mechanisms, including gap junctions, tunneling nanotubes (TNTs), and extracellular vesicles, plays a critical role in maintaining tissue homeostasis and coordinating cellular functions. Gap junctions are channels connecting adjacent cells, allowing the exchange of ions, small molecules, and regulatory RNAs. They are essential for processes like electrical signaling in the heart and metabolic coupling. However, during aging, there is a decline in gap junction function due to changes in protein levels and distribution, contributing to age-related disorders in various organs [164]. A decline in the levels of Connexin 43 (Cx43), a protein involved in gap junction formation, leads to impaired intercellular communication in various organs. For example, in the heart, reduced Cx43 levels and disrupted gap junctions result in slower electrical impulse propagation and cardiac dysfunction [165]. One study using obese rats fed with a high-fat diet showed that ginger (applied orally at 500 mg/kg body weight/day for 40 days) markedly reduced triacylglycerol, total cholesterol, LDL-C, MDA, collagen I, and MMP-2, potentially mitigating tissue damage and the progression of cardiovascular complications [166]. In bone tissue, reduced Cx43 levels contribute to increased susceptibility to oxidative stress-induced cell death and decreased bone strength. Notably, manipulating Cx43 expression can mitigate these age-related changes, indicating its significance in protecting against age-induced alterations [167]. Notably, TNF-α expression-inhibitory effects were also demonstrated in other works, for example, upon application of ginger extract in human synoviocyte cultures [168], and upon ginger extract (100 mg/Kg b.wt) oral application before gamma radiation exposure in Swiss albino mice [169].

Tunneling nanotubes are thin, dynamic membrane structures that facilitate the transfer of cellular components between distant cells. While they can promote beneficial signals and rescue cells from damage, they can also propagate harmful molecules and cellular stress. These nanotubes are increasingly recognized for their role in various physiological processes and diseases, including cancer, development, and immune responses [170]. During aging, the formation of TNTs may increase due to elevated oxidative stress and inflammation, contributing to age-related changes and disease progression. Extracellular vesicles (EVs), such as exosomes and microvesicles, are small membrane-bound particles that carry various biomolecules and can influence neighboring cells. EVs derived from senescent cells are known to be part of the senescence-associated secretory phenotype (SASP) and contribute to age-related changes. They can trigger cellular responses and spread senescence-related signals, impacting inflammation, immune responses, and tissue regeneration. The composition of EVs changes with aging, affecting their functional effects on recipient cells [164]. One research finding specifically indicates that ginger-derived exosome-like nanoparticles (EPDENs) exert notable effects on cellular behavior. When ginger EPDENs (applied at 1.0 µg/mL for 24 h) were introduced to RAW 264.7 macrophages, they distinctly induced the expression of two significant elements: antioxidation gene heme oxygenase-1 and the anti-inflammatory cytokine IL-10. This differential impact of ginger EPDENs showcases their potential to modulate key genetic factors associated with antioxidation and anti-inflammatory responses within macrophages. This insight into ginger EPDENs’ ability to influence specific gene expression highlights their unique properties and underscores their potential therapeutic value for enhancing antioxidative and anti-inflammatory cellular pathways [158].

The process of aging is characterized by deficiencies in various neural, neuroendocrine, and hormonal signaling pathways [171]. One study focused on assessing the anti-inflammatory and neuroprotective effects of 6-shogaol, a key active compound in ginger, across molecular, cellular, and in vivo contexts. The investigation centered on its potential to regulate neuroinflammation by inhibiting microglial activation in the brain. The study found that 6-shogaol exhibited anti-inflammatory properties, downregulated microglial activation, and demonstrated significant neuroprotective effects in a model of ischemia (applied at 1, 3, and 6 mg/kg). The results highlighted the potential of 6-shogaol in combating neurodegenerative diseases associated with inflammation and its possible utility as a therapeutic agent [172]. Another study showcased Fermented Ginger’s potential anti-amnesic effects against Aβ1–42 plaque toxicity by safeguarding against neuronal cell loss and synaptic disturbance. The presence of 6-paradol, stemming from the fermentation process, was identified as a potential mediator of memory improvement. The research suggests that fermented ginger prepared using *Schizosaccharomyces pombe* fermentation could offer relief from memory dysfunction and neuronal degradation associated with conditions like Alzheimer’s disease [173]. Through heterochronic parabiosis experiments and detailed single-cell transcriptomic analysis, it has been confirmed that young blood can rejuvenate various tissues [174]. On the level of molecular mediators, for example, the chemokine CCL3/MIP-1a rejuvenates hematopoietic stem and progenitor cells, the metalloproteinase inhibitor TIMP2 is implicated in hippocampal rejuvenation, and the anti-inflammatory interleukin IL-37 enhances endurance exercise and metabolic function in aged mice [175]. On this background, numerous studies demonstrated brain-specific benefits in numerous works [11]. Thus, one study showed that 6-shogaol showed improved brain function and reduced brain damage caused by ischemia, partly by impacting oxidative stress, neuroinflammation, and cell signaling pathways. The compound also protects against hippocampal cell damage [11]. 6-Paradol had a similar effect, reducing brain damage and neurological deficits. Zingerone alleviated behavioral and histological issues linked to brain ischemia by countering oxidative stress and apoptotic markers [11]. The compounds’ potential benefits were proposed to be related to their structural similarity to curcumin, which has shown protective effects in endothelial function decline and vascular atherosclerosis [176]. Taken together, while ginger and some of its bioactive compounds have demonstrated numerous presumably beneficial effects specific to the nervous system (being an important part of the body’s intercellular communication), the underlying molecular modes of action merit further research.

## 12. Potential of Ginger to Modulate Chronic Inflammation

The anti-inflammatory actions of ginger have been proven by several studies [25,177]. Inflammation has been implicated in several impactful diseases like arthritis, autoimmune diseases, cardiovascular diseases, and neurodegenerative diseases. For many centuries people have used ginger against conditions suggesting potential anti-inflammatory effects [178]. It was confirmed decades ago by researchers that ginger has an inhibitory effect on prostaglandin synthesis, and its anti-inflammatory properties resemble that of nonsteroidal anti-inflammatory drugs (NSAIDs). Along this line, ginger has an inhibitory effect on cyclooxygenase 1 and 2, and thereby, it produces an inhibitory effect on prostaglandin synthesis [41,42]. Along this line, among several ginger-derived compounds tested for COX-1 inhibition 8-paradol exhibited the strongest inhibitory activity, with an IC50 value of 4 µM [41], and among several ginger-derived compounds tested for COX-2 inhibition, the most potent was 10-shogaol, with an IC50 value of 7.5 μM [42]. Besides the above inhibitory action, ginger also suppresses 5-lipoxygenase and inhibits the synthesis of leukotrienes [177,179,180]. Thus, gingerdione, in particular, was shown to inhibit in human leukocytes 5-HETE produced by 5-lipoxygenase with an IC50 of 15 µM [179]. Chronic inflammation is an important cause of various diseases like cancer, atherosclerosis, diabetes, and rheumatoid arthritis [177,178,180,181]. Ginger and its various phytoconstituents can play a potential role in reducing inflammation. In Asian traditional medicinal preparations, ginger rhizomes have been used for the treatment of mild forms of rheumatoid arthritis and fever, and in line with such effect, ginger phenylpropanoids (gingerols, shogaols) were found to target phospholipases A2, inhibiting IL-1beta and prostanoid secretion and disrupting arachidonate-phospholipid remodeling [181].

In a study carried out on ginger extract in rats, it was found that ginger extract possessed both anticancer and anti-inflammatory properties [182]. This effect was observed by inducing hepatoma in the rats by ethionine. It was found in the study that ginger extract (100 mg/kg body weight) suppressed the increased levels of tumor necrosis factor α (TNFα) and nuclear factor kappa B (NFκB) in rats that had liver cancer thus expressing both anticancer and anti-inflammatory potential [182]. Ginger also exhibited good potential in the treatment of inflammatory bowel diseases like ulcerative colitis (UC) and Crohn’s disease (CD) [183]. Ginger and its constituents have also been shown to be effective in the context of airway inflammation, ameliorating ovalbumin-induced Th2 responses and enhancing Th1 response resulting in decreased airway inflammation in mice [71,184,185]. In the work of Khan et al. specifically, both ethanol extract (500 mg/kg) and aqueous extract (720 mg/kg) of ginger were tested (applied for 7 days) in a mouse model of ovalbumin-induced allergic asthma, and both extracts significantly inhibited Th2-mediated immune response, with reduction in goblet cell hyperplasia, infiltration of inflammatory cells in airways, edema with vascular congestion, and total and differential count of eosinophils and neutrophils in bronchoalveolar lavage fluid [71]. Also, levels of interleukin (IL)4, IL5, immunoglobulin E (IgE), and eotaxin have been found to be decreased by ginger, mediating the reduction of airway inflammation [184,186]. Ginger also regulates the functions of calcium ion channels, and by virtue of this effect, it is able to relax smooth muscles present in the airways to provide relief in symptoms of asthmatic patients [43]. In more detail, when food-grade ginger powder (0.5 mg, 1 mg, 5 mg, 10 mg, 25 mg, 50 mg, and 100 mg, suspended in 1 mL water) was applied for 30 min to human tracheas with epithelia removed and contracted with acetylcholine, a dose-dependent relaxation of the airway smooth muscle was observed [43]. Further study of the bioactive compounds of ginger identified 8-gingerol as one of the key ingredients, and when the compound (at 100 μM) was aerosolized and sprayed to naively hyperresponsive male A/J mice 15 min before challenge by methacholine it attenuated changes in central airway resistance [43]. With respect to its actions on the liver, ginger has been shown to decrease levels of interferon-gamma (INFγ) and IL6 and inhibit inflammation while also inducing improvements in pathological changes in the liver. In this context, through inhibition of NFκB activation ginger (standardized ethanoic extract, applied daily at 400 mg/kg by oral gavage, for 6 weeks) reduced the hepatic levels of several key pro-inflammatory cytokines, including IL-6, TNFα, and blunted liver pro-inflammatory responses in rats fed a high-fat diet [72]. The anti-inflammatory action of ginger is also beneficial in the case of patients suffering from diabetes. It has been found that ginger consumption shows improvements in diabetes patients by decreasing inflammatory factors like C-reactive protein (CRP), TNFα, and IL6 [123,125,184]. Along this line, Mahluji et al. conducted a randomized, double-blind, placebo-controlled trial with 64 type 2 diabetes patients randomly assigned to receive ginger (2 g per day) or placebo tablets for 2 months, and the ginger supplemented group displayed significantly lower serum TNF-α and hs-CRP levels [125]. Similarly, Arablou et al. performed a double-blinded, randomized, placebo-controlled clinical trial with 70 type 2 diabetes patients who were allocated to receive placebo or 1600 mg ginger (equaling 2 capsules) for 12 weeks, and the ginger-receiving group displayed not just significantly improved metabolic parameters (reduction of fasting glucose, HbA_1C_, insulin, HOMA, triglycerides, and total cholesterol) but also reduction in the level of the inflammatory markers CRP and PGE_2_ [123]. Through its antioxidant and anti-inflammatory effects ginger might also provide protection against arthritis, musculoskeletal disorders, and joint inflammation [180,187]. In the latter context, a study examining the effects of ginger water extract on collagen-induced arthritis (CIA) in mice and IL-1β-induced inflammation in human synovial fibroblasts [73]. The results of the study showed that ginger water extract (orally administered at 100 and 200 mg/kg body weight, once daily from day 22 to day 35 after the induction of arthritis) exerted an anti-arthritic effect by inhibiting the production in the serum of the IL-17, IL-4, and IFN –γ (interferon gamma) and the paw tissue expression of MMP (matrix metalloproteinase)-1, MMP-13, and MMP-3 in CIA mice and IL-1β-activated synovial cells were decreased [73]. Another study investigated the anti-inflammatory effect of steamed ginger extract on *Helicobacter pylori*-infected gastric (AGS) cells, that was mediated by inhibition of NF-κB [44]. The results of the study showed that the ginger extract used in the study (applied at 1–200 μg/mL for 24 h) not only inhibited the growth of *Helicobacter pylori* but also reduced in the gastric cells *Helicobacter pylori* induced inflammation markers including IL-8, TNF-α, IFN-γ, IL-6, iNOS, and myeloperoxidase [44].

Several specific phytochemicals present in ginger were demonstrated to play a role in its anti-inflammatory actions. The inhibitory effects of 6-shogaol on cell functions related to angiogenesis and inflammation were evaluated in primary endothelial cells of human origin (HUVEC) [45]. The results of the study showed that 6-shogaol (applied 30 min before LPS challenge at 1–30 µM) showed inhibitory effects on angiogenesis and inflammation in LPS-activated HUVECs, inhibiting the activation of NF-κB, the expression of pro-inflammatory adhesion molecules (ICAM-1, VCAM-1, E-selectin), and the attachment of leukocytes [45]. Another study also concluded that 6-shogaol (applied daily via gavage at 6.2 mg/kg for 28 days) was effective in decreasing chronic inflammatory response, and provided protection against damage to femoral cartilage (reflected in reduced swelling, preservation of the morphological integrity of the cartilage lining the femur, reduced leukocyte infiltration and lower concentration of soluble 1 VCAM-1) in rats who were subjected to monoarthritis in the right knee (by Freund’s Adjuvant injection) [46]. Agents that can inhibit microglial activation have the potential to counteract neurodegenerative diseases, and in this context, one study examined the effects of 6-shogaol (applied at 10 µM for up to 24 h) on microglia activation in BV-2 and primary microglial cell cultures [172]. The results of the study demonstrated that 6-shogaol proved to be an effective candidate for treating neurodegenerative diseases since it inhibited microglial activation and showed neuroprotective and anti-neuroinflammatory actions [172]. Another study showed that 6-shogaol (10 mg·kg^−1^·d^−1^, orally, for 3 days) in a mouse (C57/BL) model of Parkinson’s disease provided anti-neuroinflammatory action and neuroprotective effects to dopaminergic neurons (including reversal of the MPTP-induced changes in motor coordination and bradykinesia, and reversal of dopaminergic neuronal loss in substantia nigra pars compacta and in stratum) [60]. Anti-inflammatory activities have been also demonstrated for 6-gingerol. Thus, one study found that 6-gingerol (given once a day by gavage at 250 mg/kg for 14 days) showed beneficial effects on ulcerative colitis mice model (dextran sulfate sodium (DSS)-induced colitis) by ameliorating bowel damage and reducing incidence of weight loss, as well as suppressing the increased serum and bowel levels of cytokines such as IL-6 and IL-17 (indicating inhibition of inflammatory responses both systematically as well as locally), and affecting the cell balance of Th17/Treg [74].

## 13. Potential of Ginger to Modulate Dysbiosis

Dysbiosis can be regarded as an alteration in the composition or balance of gut microbes that has an impact on the health of a person, and new research is indicating an increasing number of diverse diseases associated with gut dysbiosis [188,189]. In this context, newer medicines that can produce a positive effect to restore the proper balance of intestinal microbes and cure dysbiosis are urgently required, and natural products can be good candidates with potential effectiveness and proven safety [190,191]. Several studies have assessed the impact of ginger on dysbiosis. In one study, the effect of a combined aqueous extract of olive leaves and ginger rhizome was assessed on gut microbiota in healthy rats as well as rats that were made diabetic with alloxan [192]. The results of the study showed that as a result of the application of the extracts (given separately or in a combination at a dose of 500 mg/kg per day, with or without insulin, for 7 days) there was a general enhancement in the bacterial diversity and an increase in the Firmicutes/Bacteroidetes ratio observed in both healthy and diabetic rats upon the administration of the combined aqueous extract. In insulin treated diabetic rats, the combined extract decreased the levels of the unfavorable *Clostridium*. The results in healthy rats showed that the administration of the combined extract caused an increase in the relative abundance of the considered as favorable *Lactobacillus* and *Prevotella* whereas there was a decrease in the relative abundance of the considered as unfavorable *Clostridium* and *Bacteroides* [192].

Research was also carried out to evaluate the effect of dried ginger rhizome powder (DGRP) to reduce obesity by modulation of gastrointestinal microbiota in mice [75]. In this study, two groups of mice were fed a high-fat diet (HFD), and two groups of mice were fed a normal chow diet (NCD). Among the HFD groups, one group received HFD only and one group received HFD along with DGRP. On the other hand, in the NCD groups, one group received only NCD, and the other group received NCD along with DGRP. Also, mice that had depleted microbiota were transplanted with the fecal microbiota of mice administered a HFD or mice administered a HFD and ginger supplementation [75]. Different parameters were studied in this work, and it was found that in mice who received HFD along with DGRP treatment (applied at 500 mg/kg body weight ginger once daily via gavage for 16 weeks), there was a decrease in low-grade inflammation, liver steatosis, and body weight. Also, improvements with respect to insulin resistance were observed in these mice. It was found that ginger supplementation produced modulation in the composition of gut microbiota with an increase in the species which belong to the genus *Bifidobacterium* and bacteria that produce short-chain fatty acids (SCFAs). Fecal concentration of SCFAs was also found to increase [75]. *Bifidobacterium* bacteria play an important role in improving metabolic health. This group of microorganisms is considered to provide protection to the body against obesity, insulin resistance, and low-grade inflammation [193,194]. SCFA-producing bacteria are also considered beneficial for health in general, with a negative correlation existing between these bacteria and obesity, inflammation, and insulin resistance [195,196,197]. Importantly, through fecal microbiota transplantation (FMT), it was demonstrated that the anti-obesity and health-promoting effects of ginger were transferrable [75].

Another study was conducted on healthy humans (crossover intervention study with 123 healthy subjects, of which 63 men and 60 women) to study the effect of a short-term intake of fresh ginger juice (20 mL freshly squeezed ginger juice per day for 7 days) on the variation of gut microbiota and its relevance to human health [126]. The results of the study demonstrated that the species number of the microbial flora of intestines was increased. An increase in the ratio of Firmicutes-to-Bacteroidetes, levels of anti-inflammatory *Faecalibacterium*, and levels of Proteobacteria was also observed [126]. Also, a decrease in relative abundance in the ratio of Prevotella-to-Bacteroides and levels of pro-inflammatory *Ruminococcus_1* and *Ruminococcus_2* was observed in the same study. Overall, this work provides evidence that ginger juice had a strong effect on the function and composition of gut microbiota in people who were healthy [126].

In the context of dysbiosis, a study was also carried out in mice (BALB/c mice) administered dextran sulfate sodium to induce ulcerative colitis [76]. In this study, the effect of ginger powder on reducing the severity of ulcerative colitis by improving the function and diversity of gut microbiota was assessed. The results of the study highlighted the fact that ginger powder (orally administered at 500 mg/kg daily for 7 days) produced a beneficial effect on ulcerative colitis by positively influencing the microbial population of the intestine [76]. Mice with colitis had lower species diversity and richness, a higher abundance of pathogenic bacteria, *Proteobacteria* and *firmicutes*, an increase in *Lachnospiraceae_NK4A136_group*, and an increase in the abundance of *Lactobacillus_murinus*, *Lachnospiraceae_bacterium_615*, and *Ruminiclostridium_sp._KB18*. These increased pathogenic bacteria were decreased by ginger intake. Similarly, the DSS-treated mice showed a lower abundance of *Muribaculaceae*, and ginger application reversed this trend [76].

The role of gut microbiota with respect to cardiovascular diseases is gaining increasing interest in modern medical science [188]. The effect of ginger essential oil (50 mg/kg bw/day or 100 mg/kg bw/day, via oral gavage, for 16 weeks) and one of its bioactive compounds, citral (20 mg/kg bw/day, via oral gavage, for 16 weeks), on atherosclerosis was evaluated in Apo E mice made atherosclerotic through administration of Gubra Amylin NASH (nonalcoholic steatohepatitis) diet with L-carnitine [77]. During the metabolism of L-carnitine, trimethylamine-N-oxide is formed which is involved in the formation of atherosclerotic plaques leading to thrombosis. The conducted study showed that citral and ginger essential oil produced improvement in resistance to insulin and plasma lipid profile; decreased levels of sugar in blood and trimethylamine-N-oxide levels; inhibited plasma levels of inflammatory cytokines like interleukin-1β, and most importantly, inhibited the formation of aortic atherosclerotic lesions in the animals used in the study [77]. Treatment with citral and the essential oils of ginger also produced a modulating effect on the function and diversity of gut microbiota. Both treatments lead to an increase in the abundance of microbes that are beneficial and a decrease in the abundance of microbes that are involved in the production of cardiovascular diseases. Thus, by positively influencing the gut microbiota, citral and ginger essential oils demonstrated a good potential recommending them as beneficial supplements to counteract cardiovascular diseases [77].

Since dysbiosis has been suggested to be implicated in a variety of diseases and ginger supplementation displayed benefits in restoring a healthy microbiota composition, the study of the potential of ginger-based supplements to combat such diseases can prove to be a highly interesting topic for researchers in the coming years.

## 14. Conclusions

Preclinical research has shown that ginger extracts and bioactive compounds are able to exhibit some favorable effects on all twelve hallmarks of aging. Particularly, numerous preclinical animal research studies have indicated positive impacts on deregulated nutrient-sensing, mitochondrial dysfunction, chronic inflammation, and dysbiosis. The reviewed research suggests that ginger might have the potential to act as a multi-targeting nutraceutical, simultaneously affecting multiple hallmarks of aging through several of its bioactive constituents and a range of diverse mechanisms. However, the present work also reveals that validation in human clinical trials is still insufficient or entirely missing, with the exception of some studies indicating benefits on deregulated nutrient-sensing, chronic inflammation, and dysbiosis. Thus, the accumulated compelling body of research evidence warrants further clinical validation studies of ginger and some of its constituents as interventions for lifespan extension and healthy aging.

## Figures and Tables

**Table 1:** In vitro studies linked to the potential of ginger to modulate hallmarks of aging.

Ginger Ingredient	Hallmark of Aging	In Vitro Model and Reference	Dose and Time of Treatment	Outcomes
6-Shogaol	Epigenetic alterations	Rat primary cultured cardiac fibroblasts [30]	Preincubation with 0.3 µM and 1 µM 6-shogaol for 2 h	Blocked phenylephrine- and TGF-β-induced acetylation of histone H3K9
6-Shogaol	Epigenetic alterations	p300-Histone acetyltransferase assay [30]	30 min incubation with 6-shogaol (0.01–100 μM)	6-shogaol inhibits p300-histone acetyltransferase dose-dependently, with IC50 value of 6.77 µM
Water extract of ginger	Epigenetic alterations	Human breast cancer cell line MDA-MB-231 [31]	The extract was applied at a dose of 10, 30, 50, 80, and 100 μg for 24, 48, and 72 h	Restoration of the expression of tumor suppressive miRNAs, miR-200c, miR-30a, and miR-128, as well as a significant decrease of miR-200C promoter methylation and increased methylation status of LINE1
Metanolic extract of ginger	Loss of proteostasis	In vitro anti-glycating and anti-aggregation test with glucose-exposed bovine serum albumin [32]	The extract was applied in the concentration range of 100–600 µg/mL for 15 days	The extract exhibited anti-glycating and anti-aggregation properties
6-Gingerol	Disabled macroautophagy	Human umbilical vein endothelial cells exposed to hydrogen peroxide [33]	Pretreatment with 10–40 µM 6-gingerol for 24 h	Promoted autophagy, which protected the cells from apoptosis induction
Zerumbone	Deregulated nutrient-sensing	Mouse 3T3-L1 preadipocytes differentiated to adipocyte-like cells [34]	Treatment with 5 or 10 μM zerumbone for 48 h	Zerumbone inhibited adipogenesis in 3T3-L1 cells, reversed the dysregulation of miR-146b as well as attenuated decrease in SIRT1 expression
6-Gingerol	Deregulated nutrient-sensing	Mouse 3T3-L1 preadipocytes differentiated to adipocyte-like cells [35]	20 μM, for 8–9 days	Increased the expression levels of PGC-1α, PRDM16, and UCP1, promoted 3T3-L1 cells browning, mitochondrial biogenesis, p-AMPK and SIRT1 expression
6-Gingerol	Mitochondrial dysfunction	L6 myoblasts [36]	50–150 µM applied up to 24 h	Enhanced AMPK α-subunit phosphorylation, PGC-1α mRNA expression, and mitochondrial content
Ginger water extract	Mitochondrial dysfunction	CTLL-2 cells [37]	2.5–10 mg/mL for 24 h	Promoted cytotoxicity of CTLL-2 against cancer cells and increased mitochondrial mass, mtDNA copy number, and ATP production
Ginger water extract	Mitochondrial dysfunction	HepG2 cells [38]	Applied at 2.5 and 5 mg/mL for 3 days	Enhanced mitochondrial mass, mtDNA copy number, ATP production, and activities of mitochondrial respiratory chain complexes
Standardized ginger extracts (GE1 and GE2) enriched with 6-gingerol and 6-shogaol (GE1 contained 6-gingerol and 6-shogaol at concentrations of 289.531 μg/mL and 15.466 μg/mL, while GE2 contained 181.257 μg/mL and 63.425 μg/mL, respectively)	Cellular senescence	Senescent (population doubling = 21) primary human myoblasts [39]	Applied at 100 or 300 μg/mL for 24 h	The treatments led to morphological changes in the senescent myoblasts, resembling the morphology of young myoblasts, and reduced the expression of senescence-associated β-galactosidase, a marker of replicative senescence
Gingerenone A	Cellular senescence	WI-38 human fibroblasts rendered senescent by exposure to ionizing radiation [40]	Applied for 48 h at 10 or 20 μM	Gingerenone A is identified as a novel senolytic compound, effectively and selectively reducing the viability of senescent cells while enhancing the expression of cleaved caspase-3, decreasing the levels of Bcl-XL, and reducing secreted levels of pro-inflammatory factors IL-6, CCL2 (MCP-1), and interferon γ-induced protein 10 (IP-10) while increasing the anti-inflammatory cytokines IL-10 and IL-13
8-Paradol	Chronic inflammation	COX-1 inhibitor assay [41]	Tested for 10 min in several concentrations (0–100 µM) for IC50 determination	Among several ginger-derived compounds, 8-paradol exhibited the strongest COX-1 inhibitory activity, with an IC50 value of 4 µM
10-Gingerol, 8-shogaol and 10-shogaol	Chronic inflammation	COX-2 inhibitor assay [42]	Each compound was tested in 12 different concentrations for IC50 determination	The three compounds inhibited COX-2 with IC50 values of 32 μM, 17.5 μM, and 7.5 μM, respectively
Food-grade ginger powder suspended in water		Human tracheas with epithelia removed contracted with acetylcholine (a model for relaxation of the airway smooth muscle) [43]	The food-grade ginger powder (0.5 mg, 1 mg, 5 mg, 10 mg, 25 mg, 50 mg, and 100 mg, suspended in 1 mL water) was applied for 30 min	A dose-dependent relaxation of the airway smooth muscle was observed
Steamed ginger extract	Chronic inflammation	Helicobacter pylori-infected gastric (AGS) cells [44]	Applied at 1–200 μg/mL for 24 h	Inhibited the growth of Helicobacter pylori, but also reduced in the gastric cells Helicobacter pylori-induced inflammation markers, including IL-8, TNF-α, IFN-γ, IL-6, iNOS, and myeloperoxidase
6-Shogaol	Chronic inflammation	LPS-challenged HUVECs [45]	Applied 30 min before LPS challenge at 1–30 µM [45]	Exhibited inhibitory effects on angiogenesis and inflammation in LPS-activated HUVECs, inhibiting the activation of NF-κB, the expression of pro-inflammatory adhesion molecules (ICAM-1, VCAM-1, E-selectin), and the attachment of leukocytes
6-Shogaol	Chronic inflammation	Rats subjected to monoarthritis in the right knee by Freund’s Adjuvant injection [46]	Applied daily via gavage at 6.2 mg/kg for 28 days	Decreased chronic inflammatory response and provided protection against damage to femoral cartilage, reflected in reduced swelling, preservation of the morphological integrity of the cartilage lining the femur, reduced leukocyte infiltration, and lower concentration of soluble 1 VCAM-1

**Table 3:** Human research studies linked to the potential of ginger to modulate hallmarks of aging.

Hallmark of Aging	Study Design and Reference	Ginger Ingredient	Dose and Time of Treatment	Outcomes
Deregulated nutrient-sensing	A double-blind, randomized, placebo-controlled trial with 70 type 2 diabetes patients [123]	Ginger capsules	1600 mg ginger (equaling two capsules) daily for 12 weeks	The ginger-receiving group displayed both significantly improved metabolic parameters (reduction of fasting glucose, HbA_1C_, insulin, HOMA, triglycerides, and total cholesterol) as well as a reduction in the level of the inflammatory markers CRP and PGE_2_
	A double-blind, randomized, placebo-controlled clinical trial with 103 type 2 diabetes patients [124]	Ginger capsules	Daily intake of 1.2 g of ginger (two capsules, 600 mg ginger powder in each) for 90 days	A greater reduction in the blood glucose and total cholesterol in comparison to the placebo group
Chronic inflammation	Randomized, double-blind, placebo-controlled trial with 64 type 2 diabetes patients [125]	Ginger tablets	2 g per day (equaling two tablets) for 2 months	The ginger supplemented group displayed significantly lower serum TNF-α and hs-CRP levels
	A double-blind, randomized, placebo-controlled trial with 70 type 2 diabetes patients [123]	Ginger capsules	1600 mg ginger (equaling two capsules) daily for 12 weeks	The ginger-receiving group displayed not just significantly improved metabolic parameters (reduction of fasting glucose, HbA_1C_, insulin, HOMA, triglycerides, and total cholesterol) but also a reduction in the level of the inflammatory markers CRP and PGE_2_
Dysbiosis	Crossover intervention study with 123 healthy subjects, of which 63 men and 60 women [126]	Freshly squeezed ginger juice	20 mL freshly squeezed ginger juice per day for 7 days	The species number of the microbial flora of intestines was increased, and also the ratio of Firmicutes-to-Bacteroidetes; levels of anti-inflammatory Faecalibacterium; and levels of Proteobacteria, while there was a decrease in relative abundance in the ratio of Prevotella-to-Bacteroides and levels of pro-inflammatory Ruminococcus_1 and Ruminococcus_2
